# Editorial: Physiology, Application, and Bioengineering of Oleaginous Microorganisms

**DOI:** 10.3389/fmicb.2021.650957

**Published:** 2021-05-14

**Authors:** Xiaochao Xiong, Yu Xia, Jianjun Qiao

**Affiliations:** ^1^Department of Biological Systems Engineering, Washington State University, Pullman, WA, United States; ^2^State Key Laboratory of Food Science and Technology, School of Food Science and Technology, Jiangnan University, Wuxi, China; ^3^Department of Pharmaceutical Engineering, School of Chemical Engineering and Technology, Tianjin University, Tianjin, China; ^4^Key Laboratory of Systems Bioengineering (Ministry of Education), Tianjin University, Tianjin, China; ^5^SynBio Research Platform, Collaborative Innovation Center of Chemical Science and Engineering (Tianjin), Tianjin, China

**Keywords:** oleaginous yeasts, *Yarrowia lipolytica*, *Rhodosporidium toruloides*, metabolic engineering, synthetic biology, lipid

## Introduction

The current Research Topic provides an effective communication platform, collecting both original research articles and review papers examining explorations of the mechanism for lipid accumulation, biotechnological applications, and metabolic engineering efforts related to oleaginous fungi including the non-conventional yeasts. Microbes have been harnessed for the production of hydrocarbon with a high-energy density as “drop-in” fuels, renewable chemicals, and value-added compounds. In addition to the commonly used model organisms such as *Escherichia coli* and *Saccharomyces cerevisiae*, over the past few years, oleaginous yeasts that naturally accumulate high-content lipids have been directly used or genetically modified for producing diverse bioproducts, although early trials on the commercial production of microbial oil date back to World War I. This Research Topic concentrates on the advancement of bioengineering of oleaginous yeasts, including *Yarrowia lipolytica* and *Rhodosporidium* (*Rhodotorula*) *toruloides*, for producing biofuels and bioproducts, with particular emphasis on the establishment of synthetic biology tools and novel engineering strategies.

## Synthetic Biology Tools for Oleaginous Yeasts

Synthetic biology facilitates the Design-Build-Test-Learn (DBTL) biological engineering cycle for strains development and improvement. The sets of molecular biology toolbox have been established for the genetic manipulation of non-conventional yeasts *Y. lipolytica* (Bredeweg et al., 2017) and *R*. *toruloides* (Park et al., 2018). As an essential genetic unit to control the expression of targeted genes, the constitutive, inducible, and repressible promoters have been cloned and characterized in both strains (Nora et al., 2019). The strength of the hybrid promoters in *Y. lipolytica* could be fine-tuned by engineering tandem copies of upstream activation sequences (UASs) (Blazeck et al., 2011; Xiong and Chen, 2020). Genetically encoded biosensors were recently developed in response to the dynamic changes of the cellular contents of malonyl-CoA and flavonoid in *Y. lipolytica* by recruiting bacterial transcriptional factors, and they were used to improve the stability and yield of the engineered strains (Lv et al., 2020). The Cre-*loxp* recombination system was devoloped for the marker-less deletion of genes and integration of DNA fragments into the genome of the strains such as *Y. lipolytica* and *R*. *toruloides* and the homologous recombination frequency could be increased in the strains with disruption of the *Ku70* encoding gene (Koh et al., 2014). The clustered regularly interspaced short palindromic repeats (CRISPR) and CRISPR-associated proteins (CRISPR-Cas) technologies have been successfully developed for the oleaginous microorganisms *Y. lipolytica* and *R*. *toruloides* to carry out genome editing (Schultz et al., 2019; Abdel-Mawgoud and Stephanopoulos, 2020; Yang et al., 2020). Furthermore, the current CRISPR-based technologies in oleaginous yeasts including *R*. *toruloides* can be optimized to achieve multiplexed genome engineering by lowering off-target effects and improving the efficiency (Jiao et al., 2019; Otoupal et al., 2019).

## Metabolic Engineering of Oleaginous Yeasts

Oleaginous microorganisms are particularly attractive as emerging microbial chassis for metabolic engineering. In oleaginous microorganisms, the metabolism naturally results in high flux through acetyl-CoA and NAPDH, the precursor and reducing power for the biosynthesis of lipids and many other bio-based products. As an organism with a status of Generally Recognized as Safe (GRAS), the oleaginous yeast *Y*. *lipolytica* has been engineered for producing microbial lipid with a titer of 99 g L^−1^ and a rate of 1.2 g/L/ h (Qiao et al., 2017). The lipid-based chemicals such as fatty alcohol, wax esters, and unusual fatty acids including ricinoleic acid (Béopoulos et al., 2014) and eicosapentaenoic acid (EPA) classified as n-3 (omega-3) polyunsaturated fatty acids (PUFA) were produced in the recombinants of *Y*. *lipolytica* by reprogramming lipid and fatty acid biosynthesis (Xue et al., 2013). The product portfolio was extended to biosynthesize organic acids such as succinic acid and the sugar substitutes such as erythritol by metabolic engineering of *Y*. *lipolytica*. In parallel, considerable progress has been made in the metabolic engineering of *R*. *toruloides* for producing both lipid-based compounds and other chemicals such as indigoidine, a blue pigment (Wehrs et al., 2019; Liu et al., 2020; Wen et al., 2020).

To construct the productive cell factories of oleaginous microorganisms, some novel metabolic engineering strategies including engineering central carbon metabolism and pathway compartmentalization have been employed. In the oleaginous yeast *Y*. *lipolytica*, NADPH to support lipid biosynthesis was primarily generated from the oxidative pentose phosphate pathway (PPP) when glucose was used as a carbon source, this resulted in carbon loss as released CO_2_ for the biosynthesis of the end product (Wasylenko et al., 2015). To re-balance the redox potential for the biosynthesis of lipid in *Y*. *lipolytica*, different synthetic pathways were engineered in yeast cytosol to convert glycolytic NADH into NADPH (Qiao et al., 2017). Acetyl-CoA generation was enhanced to improve the production of triacetic acid lactone (TAL) by engineering the pyruvate dehydrogenase (PDH) complex, pyruvate PDH bypass pathway, and β-oxidation in *Y*. *lipolytica* (Markham et al., 2018). Pathway compartmentalization leads to both high concentrations of precursor supply and high enzyme activities, alleviation of the competition from other metabolic pathways, and increases the sink capacity of the host for the accumulation of products. Other than the pathways engineered in the cytoplasm, pathway construction and modification were conducted in the endoplasmic reticulum, mitochondria, and peroxisomes of *Y*. *lipolytica* (Xu et al., 2016). Furthermore, the transport of metabolites across different organelles could be re-wired by manipulation of the corresponding transporters to redirect metabolic flux toward target biosynthesis. As an example, the production of itaconic acid biosynthesis was improved by overexpression of the gene encoding a mitochondrial tricarboxylate transporter from *Aspergillus terreus* in *Y*. *lipolytica* (Zhao et al., 2019). Another peculiar feature of oleaginous microorganisms is the formation of lipid droplets as a cellular compartment for the storage of neutral lipids including triacylglycerols (TAG) and/or sterol esters (SE). It found that there was a synergy between lipid accumulation and lipid-soluble pigments such as lycopene and β-carotene production. The titer of 6.5 g/L of β-carotene was achieved by engineering both carotenoid biosynthesis and lipid accumulation in *Y. lipolytica* (Larroude et al., 2018). By capitalizing on the uniqueness of oleaginous microbes as platform organisms, it is a promising route to develop efficient cell factories by using these advanced metabolic engineering approaches.

## Author Contributions

XX wrote the manuscript. YX and JQ provided comments and helped with the revision of the manuscript. All the authors approved the submission of this manuscript.

## Conflict of Interest

The authors declare that the research was conducted in the absence of any commercial or financial relationships that could be construed as a potential conflict of interest.
